# New quinazoline derivatives with anti-inflammatory activity and computationally predicted PDE7A binding: synthesis, molecular modeling, and *in silico* pharmacokinetic evaluation

**DOI:** 10.3389/fphar.2026.1859798

**Published:** 2026-08-21

**Authors:** Majed Alharbi, Ashwaq A. Almagati, Magdy M. Gineinah, Afaf A. El-Malah, Mohammed B. Hawsawi, Mohammad Jaffar Sadiq Mantargi, Anfal S. Aljahdali

**Affiliations:** 1 Department of Pharmaceutical Chemistry, Faculty of Pharmacy, King Abdulaziz University, Jeddah, Saudi Arabia; 2 Department of Chemistry, Faculty of Science, Umm Al-Qura University, Makkah, Saudi Arabia; 3 Department of Pharmaceutical Sciences, Pharmacy Program, Batterjee Medical College, Jeddah, Saudi Arabia

**Keywords:** anti-inflammatory activity, benzylthioquinazolinone, hydrazone, molecular docking, molecular dynamics, phosphodiesterase 7A, quinazoline

## Abstract

Quinazoline scaffolds are widely recognized in drug discovery for their diverse pharmacological profiles, and several quinazoline derivatives have been reported as phosphodiesterase 7A (PDE7A) inhibitors with potential anti-inflammatory effects. This study aimed to synthesize novel quinazoline derivatives, evaluate their anti-inflammatory activity and investigate their computationally predicted binding to PDE7A. A series of quinazoline hydrazone (6a–j) and benzylthioquinazolinone (7a–c, 8a,b) derivatives were synthesized and structurally characterized using IR, ^1^H/^13^C NMR, mass spectrometry, and elemental analysis. Anti-inflammatory activity was evaluated *in vivo* using the carrageenan-induced rat paw edema model. An acute oral toxicity assessment identified the highest tested doses without observed mortality or overt clinical toxicity of 1000–1200 mg/kg, from which anti-inflammatory test doses of 100–120 mg/kg were selected. Compound 6f produced the highest numerical inhibition among the synthesized derivatives, with 79.39% inhibition at 100 mg/kg. Compounds 6a, 6b, and 8a produced 61.85%, 61.65%, and 60.12% inhibition, respectively, at 120 mg/kg, whereas diclofenac produced 68.21% inhibition at 7.5 mg/kg. Molecular docking, Prime MM–GBSA calculations, and 100-ns molecular dynamics simulations were used to evaluate predicted PDE7A interactions and comparative dynamic behavior. Compound 6f showed the most favorable Glide GScore and IFDScore, persistent Tyr211 and Gln413 hydrogen bonds, and the clearest apparent late-stage stationary root-mean-square deviation regime. In silico ADMET analysis indicated acceptable predicted drug-likeness and absorption-related properties, although moderate-to-low aqueous solubility and predicted hERG liability require experimental evaluation. Overall, compound 6f was prioritized as the leading anti-inflammatory candidate within the evaluated series; however, PDE7A involvement remains computationally inferred and requires direct biochemical and selectivity validation.

## 1 Introduction

Quinazoline is a bicyclic azaheterocycle that has emerged as a versatile scaffold in medicinal chemistry (Bonnaud et al., 2024). Quinazoline derivatives exhibit a wide range of biological activities, including anti-inflammatory, anticancer, antimicrobial, anticonvulsant, antihyperlipidemic, antihypertensive, antimalarial, and antidepressant effects, owing to their favorable interactions with multiple molecular targets (Kurogi et al., 1996; Na et al., 2008; Zhu et al., 2010; Redondo et al., 2012; Desai et al., 2013; Al-Salem et al., 2015; Venkatesh et al., 2015; Zhang et al., 2015; Alaa et al., 2016; Zhou et al., 2024). Among the key mechanisms underlying their pharmacological utility, inhibition of phosphodiesterases (PDEs), particularly cAMP-specific PDE isoforms, plays a major role in anti-inflammatory activity (Paterniti et al., 2011; Shah et al., 2020).

Phosphodiesterases constitute a large superfamily of phosphohydrolases (PDE1–PDE11) that regulate intracellular cyclic nucleotides, mainly cAMP and cGMP (Shah et al., 2020). PDE4, PDE7, and PDE8 preferentially hydrolyze cAMP, whereas PDE5, PDE6, and PDE9 are selective for cGMP, and the remaining families show dual specificity (Shah et al., 2020). Elevation of intracellular cAMP exerts immunosuppressive and anti-inflammatory effects; however, early generations of nonselective or PDE4-selective inhibitors were associated with dose-limiting gastrointestinal adverse effects. A promising strategy to overcome these limitations is the selective targeting of PDE7, particularly PDE7A, which is predominantly expressed in inflammatory and immune cells and plays a key role in regulating pro- and anti-inflammatory mediators as well as T-cell proliferation (Paterniti et al., 2011; Elfeky et al., 2019; Świerczek et al., 2021).

Several quinazoline-based PDE7 inhibitors have been reported, demonstrating neuroprotective and anti-inflammatory effects with improved tolerability profiles compared with earlier PDE inhibitors (Elfeky et al., 2019). Nevertheless, there remains a need for new quinazoline derivatives that combine potent anti-inflammatory activity, favorable PDE7A binding characteristics, and favorable physicochemical and pharmacokinetic properties.

Guided by these considerations and previous reports on quinazolinone and quinazolinethione PDE7 ligands, this work aimed to generate novel quinazoline derivatives incorporating hydrazone and benzylthio moieties to enhance predicted interactions within the PDE7A catalytic pocket.

The study reports the synthesis and structural characterization of a series of 4-hydrazonoquinazoline derivatives (6a–j) and benzylthioquinazolin-4-ones derivatives (7a–c and 8a,b), together with their *in vivo* anti-inflammatory evaluation. Molecular docking and 100-ns molecular dynamics (MD) simulations were employed to investigate the predicted binding modes and comparative dynamic behavior of selected biologically active compounds within the PDE7A catalytic site. In addition, *in silico* ADMET profiling was performed to evaluate the predicted pharmacokinetic and safety-related properties of the prioritized compounds. The overall objective was to identify promising anti-inflammatory lead compounds with computationally predicted PDE7A binding and to provide a structural basis for subsequent optimization and direct biochemical validation.

## 2 Materials and methods

### 2.1 Chemistry

Sigma-Aldrich Company is the only supplier of all the chemicals used. Melting points were determined using a Griffin apparatus and are uncorrected. IR spectra were recorded on a Shimadzu IR 435 spectrophotometer (Shimadzu Corp., Kyoto, Japan), Faculty of Pharmacy, Cairo University, Cairo, Egypt, and values were represented in cm^-1^. Microanalyses for C, H, and N were carried out at the Regional Center for Mycology and Biotechnology, Faculty of Pharmacy, Al-Azhar University, and the values were accepted within a range of ±0.4% of the theoretically calculated percentages. In addition, Mass spectra were recorded on Jeol-Gas chromatography-mass spectrometry, JMS-Q1500GC (Tokyo, Japan), controlled with Escrime software. ^1^HNMR spectra were carried out on a Bruker 400 MHz (Bruker Corp., Billerica, MA, USA) spectrometer, Faculty of Pharmacy, Cairo University, Cairo, Egypt. Chemical shifts were recorded in ppm on the *δ* scale, coupling constants (*J*) were given in Hz, and peak multiplicities are designated as follows: s, singlet; d, doublet; dd, doublet of doublet; t, triplet; m, multiplet. ^13^CNMR spectra were recorded on a Bruker 100 MHz spectrometer at the Faculty of Pharmacy, Cairo University, Cairo, Egypt. The progress of the reactions was monitored by TLC using a precoated aluminium sheet of silica gel MERCK 60 F 254 and was visualized under UV light. Compounds 2a-c and 3a-c were prepared according to the reported procedures (Russo and Ghelardoni, 1967). Compounds 4a-c were prepared according to the reported procedure (Curd, Landquist, and Rose, 1947; El-Kerdawy et al., 1990).

#### 2.1.1 General procedure for the preparation of compounds 5a-c

Anhydrous hydrazine (0.6 g, 19 mmol) was added to a solution of the appropriate dichloroquinazoline derivative 4a-c (2.5 mmol) in ethanol (20 mL) at 0 °C. The reaction mixture was allowed to stir for 30 min at the same temperature. The product was collected by filtration, washed with ethanol, and recrystallized from EtOH/CH_2_Cl_2_.

#### 2.1.2 General procedure for the preparation of compounds 6a-j

A mixture of quinazolylhydrazine derivative 5a-c (1.54 mmol), the proper aldehyde (1.62 mmol), and absolute ethanol (25 mL) was allowed to stir at room temperature for 30 min. The product was collected by filtration and recrystallized from acetonitrile (El-Kerdawy et al., 1990).

#### 2.1.3 2-Chloro-6-nitro-4-[2-(3-pyridylmethylene)hydrazino]quinazoline (6a)

Yield, 46%; mp 277–279 °C; IR (KBr) cm^-1^: 3325 (NH), 3066 (Ar-CH), 1604 (C=N), 1581 (C=C), 1512, 1334 (NO_2_); ^1^HNMR (DMSO-d6) ppm δ: 11.74 (d, 1H, NH, D_2_O exchangeable), 9.02 (s, 1H, Ar-H), 8.78 (s, 1H, Ar-H), 8.70–8.57 (m, 3H, Ar-H), 8.27 (d, 1H, Ar-CH = N), 7.78–7.29 (m, 2H, Ar-H); ^13^CNMR (DMSO-d6) ppm δ: 167.6, 160.1 (2C), 152.4 (2C), 150.3 (2C), 135.3 (2C), 129.9 (2C), 124.6 (3C); MS m/z (%): 328 (M^+^, 21), 69 (100). Calcd. For C_14_H_9_ClN_6_O_2_ (328.72): C, 51.15; H, 2.76; N, 25.57. Found: C, 51.34; H, 2.96; N, 25.73.

#### 2.1.4 2-Chloro-4-[2-(3-chloro-2-pyridylmethylene)hydrazino]-6-nitroquinazoline (6b)

Yield, 84%; mp 248–250 °C; IR (KBr) cm^-1^: 3321 (NH), 3066 (Ar-CH), 1631 (C=N), 1573 (C=C), 1504, 1334 (NO_2_); ^1^HNMR (DMSO-d6) ppm δ: 11.74 (d, 1H, NH, D_2_O exchangeable), 9.12–9.06 (m, 1H, Ar-H), 8.55–8.27 (m, 2H, Ar-H), 7.71–7.30 (m, 3H, Ar-H), 6.90 (d, 1H, Ar-CH = N); ^13^CNMR (DMSO-d6) ppm δ: 150.8 (2C), 129.2 (3C), 123.6 (3C), 117.4 (3C), 114.8 (3C); MS m/z (%): 363 (M^+^, 15), 264 (100). Calcd. For C_14_H_8_Cl_2_N_6_O_2_ (363.16): C, 46.30; H, 2.22; N, 23.14. Found: C, 46.49; H, 2.35; N, 23.08.

#### 2.1.5 4-[2-(5-Bromo-1H-3-indolylmethylene)hydrazino]-2-chloro-6-nitroquinazoline (6c)

Yield, 39%; mp 269–271 °C; IR (KBr) cm^-1^: 3332 (NH), 3069 (Ar-CH), 1606 (C=N), 1581 (C=C), 1504, 1334 (NO_2_); ^1^HNMR (DMSO-d6) ppm δ: 11.89 (s, 1H, NH, D_2_O exchangeable), 11.70 (d, 1H, NH, D_2_O exchangeable), 8.89 (s, 1H, Ar-H), 8.48–8.46 (m, 2H, Ar-H), 7.98–7.97 (m, 1H, Ar-H), 7.45 (d, 1H, Ar-CH = N), 7.35–7.32 (m, 2H, Ar-H), 6.95–6.93 (m, 1H, Ar-H); ^13^CNMR (DMSO-d6) ppm δ: 155.7, 136.3 (2C), 133.8 (2C), 126.8, 125.6, 124.6, 117.1, 114.5 (2C), 114.2 (2C), 113.7 (2C), 112.0 (2C); MS m/z (%): 443 (M^+^, 12), 366 (100). Calcd. For C_17_H_10_BrClN_6_O_2_ (443.97): C, 45.82; H, 2.26; N, 18.86. Found: C, 46.04; H, 2.37; N, 19.12.

#### 2.1.6 2-Chloro-4-[2-(2-methyl-1H-3-indolylmethylene)hydrazino]-6-nitroquinazoline (6d)

Yield, 53%; mp 274–276 °C; IR (KBr) cm^-1^: 3317 (NH), 3069 (Ar-CH), 2920 (Aliph-CH), 1604 (C=N), 1573 (C=C), 1504, 1334 (NO_2_); ^1^HNMR (DMSO-d6) ppm δ: 11.54 (s, 1H, NH, D_2_O exchangeable), 8.92 (s, 1H, Ar-H), 8.31–8.29 (m, 2H, Ar-H), 7.35–7.33 (m, 2H, Ar-H), 7.14–7.09 (m, 3H, Ar-H and Ar-CH = N), 3.07 (s, 1H, NH, D_2_O exchangeable), 2.61 (s, 3H, CH_3_); ^13^CNMR (DMSO-d6) ppm δ: 154.2 (2C), 141.8 (2C), 136.2 (2C), 126.3 (2C), 122.1 (2C), 121.9, 120.7 (2C), 111.2 (2C), 108.3 (2C), 12.0; MS m/z (%): 380 (M^+^, 38), 67 (100). Calcd. For C_18_H_13_ClN_6_O_2_ (380.79): C, 56.78; H, 3.44; N, 22.07. Found: C, 57.03; H, 3.60; N, 22.31.

#### 2.1.7 2-Chloro-4-[2-(2-naphthylmethylene)hydrazino]-6-nitroquinazoline (6e)

Yield, 66%; mp 239–241 °C; IR (KBr) cm^-1^: 3350 (NH), 3055 (Ar-CH), 1616 (C=N), 1581 (C=C), 1504, 1334 (NO_2_); ^1^HNMR (DMSO-d6) ppm δ: 11.80 (s, 1H, NH, D_2_O exchangeable), 8.91 (s, 1H, Ar-H), 8.36 (s, 1H, Ar-H), 8.10 (d, 1H, Ar-H), 8.04–7.98 (m, 5H, Ar-H), 7.64–7.56 (m, 3H, Ar-H and Ar-CH = N); ^13^CNMR (DMSO-d6) ppm δ: 162.1 (2C), 134.8 (2C), 133.2 (2C), 132.0 (2C), 131.4 (2C), 129.1, 129.0, 128.9, 128.3, 128.2, 127.4 (2C), 125.3, 123.7; MS m/z (%): 377 (M^+^, 38), 136 (100). Calcd. For C_19_H_12_ClN_5_O_2_ (377.79): C, 60.41; H, 3.20; N, 18.54. Found: C, 60.63; H, 3.41; N, 18.72.

#### 2.1.8 6-Bromo-2-chloro-4-[2-(3-pyridylmethylene)hydrazino]quinazoline (6f)

Yield, 39%; mp 260–262 °C; IR (KBr) cm^-1^: 3194 (NH), 3066 (Ar-CH), 1616 (C=N), 1554 (C=C); ^1^HNMR (DMSO-d6) ppm δ: 11.36 (d, 1H, NH, D_2_O exchangeable), 8.93 (s, 1H, Ar-H), 8.80–8.65 (m, 1H, Ar-H), 8.04–8.01 (m, 1H, Ar-H), 7.94 (d, *J* = 2 Hz, 1H, Ar-H), 7.79 (dd, *J* = 8, 2 Hz, 1H, Ar-H), 7.68 (d, *J* = 8 Hz, 1H, Ar-H), 7.56–7.53 (m, 1H, Ar-H), 7.12 (d, 1H, Ar-CH = N); ^13^CNMR (DMSO-d6) ppm δ: 162.1, 151.4, 150.4, 140.5, 137.9, 137.5, 134.1, 130.3, 129.4, 129.3, 124.6, 118.2, 116.6, 114.2; MS m/z (%): 362 (M^+^, 13), 170 (100). Calcd. For C_14_H_9_BrClN_5_ (362.62): C, 46.37; H, 2.50; N, 19.31. Found: C, 46.51; H, 2.63; N, 19.59.

#### 2.1.9 6-Bromo-4-[2-(5-bromo-1H-3-indolylmethylene)hydrazino]-2-chloroquinazoline (6g)

Yield, 33%; mp 278–280 °C; IR (KBr) cm^-1^: 3356, 3194 (NH), 3097 (Ar-CH), 2920 (Aliph-CH), 1612 (C=N), 1512 (C=C); ^1^HNMR (DMSO-d6) ppm δ: 11.37 (d, 1H, NH, D_2_O exchangeable), 9.93 (s, 1H, NH, D_2_O exchangeable), 7.92–7.91 (m, 1H, Ar-H), 7.74–7.73 (m, 3H, Ar-H), 7.47–7.33 (m, 3H, Ar-H), 7.11 (d, 1H, Ar-CH = N); ^13^CNMR (DMSO-d6) ppm δ: 162.1 (2C), 150.4 (2C), 140.5 (2C), 137.9 (2C), 129.3 (3C), 118.2 (2C), 116.6 (2C), 114.2 (2C); MS m/z (%): 479 (M^+^, 22), 427 (100). Calcd. For C_17_H_10_Br_2_ClN_5_ (479.56): C, 42.58; H, 2.10; N, 14.60. Found: C, 42.77; H, 2.18; N, 14.86.

#### 2.1.10 6-Bromo-2-chloro-4-[2-(2-methyl-1H-3-indolylmethyleno)hydrazino]quinazoline (6 h)

Yield, 31%; mp > 280 °C; IR (KBr) cm^-1^: 3194 (NH), 3066 (Ar-CH), 2924 (Aliph-CH), 1616 (C=N), 1523 (C=C); ^1^HNMR (DMSO-d6) ppm δ: 11.36 (d, 1H, NH, D_2_O exchangeable), 10.05 (s, 1H, NH, D_2_O exchangeable), 7.94 (s, 1H, Ar-H), 7.80–7.77 (m, 2H, Ar-H), 7.60 (d, 1H, Ar-CH = N), 7.38–7.35 (m, 2H, Ar-H), 7.15–7.11 (m, 2H, Ar-H), 2.68 (s, 3H, CH_3_); ^13^CNMR (DMSO-d6) ppm δ: 162.1 (2C), 150.4 (2C), 140.5 (2C), 137.9 (2C), 129.3 (3C), 118.2 (2C), 116.6 (2C), 114.2 (2C), 12.0; MS m/z (%): 414 (M^+^, 7), 85 (100). Calcd. For C_18_H_13_BrClN_5_ (414.69): C, 52.13; H, 3.16; N, 16.89. Found: C, 52.40; H, 3.29; N, 17.05.

#### 2.1.11 2-Chloro-6-methoxy-4-[2-(3-pyridylmethylene)hydrazine]quinazoline (6i)

Yield, 34%; mp > 280 °C; IR (KBr) cm^-1^: 3182 (NH), 3066 (Ar-CH), 2939 (Aliph-CH), 1685 (C=N), 1516 (C=C); ^1^HNMR (DMSO-d6) ppm δ: 11.29 (s, 1H, NH, D_2_O exchangeable), 9.03 (s, 1H, Ar-H), 8.80–8.71 (m, 1H, Ar-H), 8.27 (s, 1H, Ar-H), 7.55–7.46 (m, 2H, Ar-H), 7.29–7.13 (m, 3H, Ar-H and Ar-CH = N), 3.87 (s, 3H, OCH_3_); ^13^CNMR (DMSO-d6) ppm δ: 163.2 (2C), 150.4 (2C), 146.7 (2C), 131.5 (2C), 122.7 (2C), 118.3 (2C), 115.9, 115.4, 56.6; MS m/z (%): 313 (M^+^, 8), 253 (100). Calcd. For C_15_H_12_ClN_5_O (313.75): C, 57.42; H, 3.86; N, 22.32. Found: C, 57.70; H, 4.05; N, 22.18.

#### 2.1.12 2-Chloro-6-methoxy-4-[2-(2-methyl-1H-3-indolylmethylene)hydrazino]quinazoline (6j)

Yield, 54%; mp 251–253 °C; IR (KBr) cm^-1^: 3313 (NH), 3093 (Ar-CH), 2974–2916 (Aliph-CH), 1612 (C=N), 1577 (C=C); ^1^HNMR (DMSO-d6) ppm δ: 11.42 (d, 1H, NH, D_2_O exchangeable), 10.50 (s, 1H, NH, D_2_O exchangeable), 8.93 (s, 1H, Ar-H), 8.32 (s, 1H, Ar-H), 7.47–7.13 (m, 6H, Ar-H and Ar-CH = N), 3.87 (s, 3H, OCH_3_), 2.61 (s, 3H, CH_3_); ^13^CNMR (DMSO-d6) ppm δ: 163.2, 154.2, 150.4, 146.6, 141.8, 136.2, 131.5, 126.3, 122.7, 122.1, 121.9, 120.7, 118.3, 115.9, 115.4, 111.2, 108.3, 56.6, 12.1; MS m/z (%): 365 (M^+^, 37), 339 (100). Calcd. For C_19_H_16_ClN_5_O (365.82): C, 62.38; H, 4.41; N, 19.14. Found: C, 62.21; H, 4.52; N, 19.36.

#### 2.1.13 General procedure for preparation of compounds 7a-c and 8a,b

A mixture of the proper substituted thioxoquinazolin-4(1*H*)-ones 3a-c (0.01 mol), substituted benzyl bromide (0.01 mol), potassium carbonate (13.8 g, 0.01 mol), and dimethylformamide (DMF) (10 mL) was heated under reflux for 12 h. The reaction was monitored with TLC until no starting materials were detected. Potassium carbonate was filtered off while hot, the remaining solution was concentrated under vacuum, and the concentrate was poured on crushed ice. The product was collected by filtration and recrystallized from aqueous ethanol.

#### 2.1.14 4-(4-Hydroxyquinazolin-2-yl)thiomethylbenzonitrile (7a)

Yield, 30%; mp 141–143 °C; IR (KBr) cm^-1^: 3325, 3213 (OH, NH), 3062 (Ar-CH), 2924 (Aliph-CH), 2225 (CN), 1604 (C=N), 1531 (C=C); ^1^HNMR (DMSO-d6) ppm δ: 8.03–7.48 (m, 3H, Ar-H), 7.40–7.12 (m, 3H, Ar-H), 6.57–6.48 (m, 2H, Ar-H), 4.99 (br.s, 1H, OH, D_2_O exchangeable), 2.97 (s, 2H, CH_2_); ^13^CNMR (DMSO-d6) ppm δ: 147.4, 144.3, 141.5, 133.1, 132.6, 130.9, 130.0, 129.7, 128.0, 127.2, 120.8, 119.4, 114.3, 109.3, 108.7, 36.5; MS m/z (%): 293 (M^+^, 8), 77 (100). Calcd. For C_16_H_11_N_3_OS (293.34): C, 65.51; H, 3.78; N, 14.32. Found: C, 65.42; H, 4.01; N, 14.46.

#### 2.1.15 4-(4-Hydroxy-6-nitroquinazolin-2-yl)thiomethylbenzonitrile (7b)

Yield, 47%; mp 159–161 °C; IR (KBr) cm^-1^: 3348, 3209 (OH, NH), 3070 (Ar-CH), 2924 (Aliph-CH), 2225 (CN), 1678 (C=N), 1604 (C=C), 1504, 1330 (NO_2_); ^1^HNMR (DMSO-d6) ppm δ: 8.08 (s, 1H, Ar-H), 7.88–7.82 (m, 2H, Ar-H), 7.71 (d, *J* = 8 Hz, 1H, Ar-H), 7.46 (d, *J* = 8 Hz, 1H, Ar-H), 7.37 (d, *J* = 8 Hz, 1H, Ar-H), 7.20 (d, *J* = 8 Hz, 1H, Ar-H), 4.88, 4.83 (2s, 1H, (OH, NH taut.), D_2_O exchangeable), 2.89 (s, 2H, CH_2_); ^13^CNMR (DMSO-d6) ppm δ: 196.8, 147.6, 141.5, 139.5, 137.3, 133.2, 132.8, 130.0, 128.0, 126.9, 118.9, 112.8, 111.1, 110.7, 44.2; MS m/z (%): 338 (M^+^, 18), 132 (100). Calcd. For C_16_H_10_N_4_O_3_S (338.34): C, 56.80; H, 2.98; N, 16.56. Found: C, 57.04; H, 3.15; N, 16.73.

#### 2.1.16 4-(6-Chloro-4-hydroxyquinazolin-2-yl)thiomethylbenzonitrile (7c)

Yield, 60%; mp 109–111 °C; IR (KBr) cm^-1^: 3313, 3155 (OH, NH), 3066 (Ar-CH), 2924 (Aliph-CH), 2225 (CN), 1670 (C=N), 1581 (C=C); ^1^HNMR (DMSO-d6) ppm δ: 7.95–7.66 (m, 2H, Ar-H), 7.56–7.06 (m, 4H, Ar-H), 6.60–6.53 (m, 1H, Ar-H), 4.81, (s, 1H, OH, D_2_O exchangeable), 2.88 (s, 2H, CH_2_); ^13^CNMR (DMSO-d6) ppm δ: 145.47, 139.1, 133.1, 132.7, 131.6, 130.3, 129.3, 128.1, 127.0, 125.4, 121.1, 119.2, 115.6, 114.1, 110.2, 46.4; MS m/z (%): 327 (M^+^, 20), 263 (100). Calcd. For C_16_H_10_ClN_3_OS (327.79): C, 58.63; H, 3.08; N, 12.82. Found: C, 58.90; H, 3.21; N, 13.09.

#### 2.1.17 2-(4-Bromo-2-fluorobenzylthio)-6-nitroquinazolin-4-ol (8a)

Yield, 73%; mp 168–170 °C; IR (KBr) cm^-1^: 3325, 3178 (OH, NH), 3066 (Ar-CH), 2920 (Aliph-CH), 1670 (C=N), 1604 (C=C), 1485, 1330 (NO_2_); 1HNMR (DMSO-d6) ppm δ: 8.63–8.56 (m, 2H, Ar-H), 8.44–8.42 (m, 2H, Ar-H), 8.31–8.27 (m, 1H, Ar-H), 8.00–7.93 (m, 1H, Ar-H), 6.01 (s, 1H, OH, D_2_O exchangeable), 2.89 (s, 2H, CH_2_); ^13^CNMR (DMSO-d6) ppm δ: 161.9, 155.3, 154.4, 150.6, 142.2, 140.9,130.5, 128.8, 127.9, 126.8, 125.2, 123.1, 119.3, 112.8, 45.1; MS m/z (%): 410 (M^+^, 37), 163 (100). Calcd. For C_15_H_9_BrFN_3_O_3_S (410.22): C, 43.92; H, 2.21; N, 10.24. Found: C, 44.17; H, 2.32; N, 10.39.

#### 2.1.18 2-(4-Bromo-2-fluorobenzylthio)-6-chloroquinazolin-4-ol (8 b)

Yield, 37%; mp 132–134 °C; IR (KBr) cm^-1^: 3394, 3267 (OH, NH), 3062 (Ar-CH), 2920 (Aliph-CH), 1670 (C=N), 1561 (C=C); ^1^HNMR (DMSO-d6) ppm δ: 7.87–7.86 (m, 1H, Ar-H), 7.65–7.60 (m, 2H, Ar-H), 7.38–7.21 (m, 2H, Ar-H), 7.09–6.96 (m, 1H, Ar-H), 5.07 (s, 1H, OH, D_2_O exchangeable), 2.73 (s, 2H, CH_2_); ^13^CNMR (DMSO-d6) ppm δ: 134.8, 128.8, 127.0 (2C), 125.6 (2C), 124.3 (2C), 121.2, 119.0 (2C), 117.3, 117.0, 116.5, 43.0; MS m/z (%): 399 (M^+^, 31), 73 (100). Calcd. For C_15_H_9_BrClFN_2_OS (399.66): C, 45.08; H, 2.27; N, 7.01. Found: C, 45.29; H, 2.41; N, 7.24.

### 2.2 Acute oral toxicity and *In vivo* anti-inflammatory activity

Albino mice of either sex weighing 25–30 g were used for the acute oral toxicity study. Test animals were obtained commercially and acclimatized under controlled laboratory conditions, including a 12 h light/12 h dark cycle, a room temperature of 25 °C ± 2 °C, and free access to a standardized commercial diet and purified drinking water.

The acute oral toxicity assessment employed a sequential small-group dosing approach informed by OECD Test Guideline 423. Mice were randomly assigned to groups of three. Before administration of each compound, the animals were housed individually and fasted for at least 8 h. The compounds were administered orally at sequential dose levels beginning at 1000 mg/kg and increasing to 1200 mg/kg or until the highest tolerated dose range was reached.

Immediately after administration, the animals were continuously observed for 4 h and periodically monitored during the subsequent 24 h. Each animal was then examined daily for 14 days for signs of toxicity, abnormal behaviour, morbidity, or mortality. Predefined humane endpoints included severe distress, seizure activity, marked respiratory difficulty, and complete loss of food/water intake. Animals reaching any of these endpoints were euthanized to minimize unnecessary suffering.

No formal statistical LD_50_ determination, such as probit analysis, was performed. Accordingly, the highest tested doses not associated with mortality or overt clinical toxicity were considered preliminary oral tolerability estimates rather than definitive LD_50_ values. One-tenth of the corresponding highest non-toxic tested dose was selected for subsequent anti-inflammatory evaluation using the carrageenan-induced paw-edema model.

Anti-inflammatory activity was evaluated using the carrageenan-induced paw edema model in 18 groups of six rats each weighing 150–180 g. The experimental groups comprised a normal control group, a carrageenan-treated disease-control group, a diclofenac-treated reference group, and groups treated with the synthesized compounds. The normal control group received distilled water without carrageenan challenge, whereas the disease-control group received distilled water followed by carrageenan administration. The synthesized compounds were administered orally at either 100 or 120 mg/kg, according to the selected dose for each compound, whereas diclofenac was administered orally at 7.5 mg/kg. All treatments and the corresponding vehicle were administered 60 min before carrageenan injection.

The test preparations were coded before administration, and paw-volume measurements were performed in a blinded manner to reduce investigator bias (Walum, 1998; Wang et al., 2015; Canadian Center for Occupational Health and Safety, 2018). Paw volume was measured at baseline and at 1, 2, and 3 h after carrageenan administration. Percentage inhibition of paw edema was calculated using the following equation:

$$\%\text{ inhibition}=\left[\left(\text{Vc }-\text{ Vt}\right)/\text{Vc}\right]\times 100$$

where Vc represents the mean paw-edema increase in the disease-control group and Vt represents the mean paw-edema increase in the treated group.

Data are expressed as mean ± SEM and were analyzed using one-way ANOVA followed by Dunnett’s multiple comparison test using GraphPad Prism (version 9.5.1). Statistical comparisons were made against the disease-control and diclofenac-treated groups. Differences were considered statistically significant at p ≤ 0.05, highly significant at p ≤ 0.01, and extremely significant at p ≤ 0.001 (Festing, 2002; Gosselin, 2019).

All experimental procedures were approved by the Research Ethics Committee of BMC University (Approval No. RES-2025–0048). Animals of both sexes were randomly allocated to the experimental groups without planned balancing by sex. Sex-dependent effects were therefore not evaluated as an independent variable in the statistical analysis.

### 2.3 Molecular modeling

#### 2.3.1 Protein and ligand preparation

The crystal structure of the catalytic domain of PDE7A in complex with 3-(2,6-difluorophenyl)-2-methylthioquinazolin-4(3H)-one (PDB ID: 3G3N) was retrieved from the Protein Data Bank (Castaño et al., 2009). Protein preparation was performed using the Protein Preparation Wizard in the Schrödinger Suite (Schrödinger Release: Protein Preparation Wizard, 2024–3; Johnston et al., 2023). The preparation protocol included the addition of hydrogen atoms, assignment of protonation states using PROPKA at physiological pH, removal of water molecules located more than 5 Å from the binding site, and energy minimization using the OPLS4 force field (Bas et al., 2008; Madhavi Sastry et al., 2013; Lu et al., 2021).

Selected biologically active compounds (6f, 6a, 6b, and 8a) were prepared using LigPrep (Schródinger Release: LigPrep 2024–3, 2024) to generate energetically minimized three-dimensional structures. Relevant protonation states, ionization states, and tautomeric forms at pH 7.4 ± 2.0 were generated using Epik, and the structures were optimized using the OPLS4 force field. The desalt option was applied during ligand preparation (Bas et al., 2008; Madhavi Sastry et al., 2013; Lu et al., 2021).

#### 2.3.2 Molecular docking

Molecular docking was performed using the Induced Fit Docking (IFD) workflow implemented in the Schrödinger Suite (Schrödinger Release 2024-3: Glide, 2024). The receptor grid was generated by centering on the co-crystallized ligand using a cubic box of 10 Å per side to define the binding region.

The IFD protocol consisted of an initial Glide docking step, followed by Prime-based refinement of side chains and nearby backbone residues within the binding site, and subsequent re-docking of the ligands into the refined receptor structures. The final docking poses were evaluated using IFDScore, Glide GScore, and Prime energy (Farid et al., 2006; Sherman et al., 2006). Binding free energies (ΔG_bind) were further estimated using the Prime MM–GBSA method (Schrödinger Release 2024-3: Prime, 2024; Hou et al., 2011; Mohamed et al., 2023).

The docking protocol was validated by re-docking the co-crystallized ligand into the active site, reproducing the experimental binding pose with a root-mean-square deviation (RMSD) of 1.69 Å (acceptable threshold <2.0 Å), confirming the reliability of the docking procedure.

#### 2.3.3 Molecular dynamics simulation

Molecular dynamics (MD) simulations were performed using Desmond in the Schrödinger Suite (Schródinger Release 2024-3: Desmond Molecular Dynamics System, D. E. Shaw Research, 2024) for the selected biologically active compounds (6f, 6b, 6a, and 8a), along with the co-crystallized ligand as a reference. Each protein–ligand complex was embedded in an orthorhombic box of TIP3P water molecules with a 10 Å buffer distance from the protein surface and neutralized by the addition of appropriate Na^+^ and Cl^−^ counterions.

The systems were subjected to 100-ns production runs under NPT ensemble conditions at 300 K and 1.01325 bar using the default Desmond relaxation protocol (Kadaoluwa Pathirannahalage et al., 2021).

Trajectory analyses included protein Cα RMSD, ligand heavy-atom RMSD calculated relative to the aligned protein, protein Cα RMSF, block-wise RMSD analysis over five sequential 20-ns intervals, and residue-specific protein–ligand interaction occupancies and timelines.

### 2.4 In silico ADMET prediction

The pharmacokinetic properties of the selected biologically active compounds (6f, 6a, 6b, and 8a) were predicted using the QikProp module implemented in the Schrödinger Suite (Schrödinger Release 2024-3: QikProp, 2024). Ligand structures were prepared using LigPrep and subjected to ADMET prediction using the default QikProp settings.

Key descriptors related to drug-likeness, physicochemical properties, absorption, distribution, and safety were selected for analysis, including molecular weight (mol_MW), lipophilicity (QPlogPo/w), aqueous solubility (QPlogS), Caco-2 permeability (QPPCaco), MDCK permeability (QPPMDCK), blood–brain barrier partition coefficient (QPlogBB), human oral absorption, and predicted hERG channel inhibition (QPlogHERG).

All calculated parameters were compared with the recommended ranges provided by QikProp to assess compliance with established drug-likeness criteria.

## 3 Results

### 3.1 Synthesis and structural characterization

The quinazoline derivatives were synthesized through two distinct synthetic routes. The first route involved the preparation of hydrazone derivatives 6a-j, initiated from substituted 2,4-dioxoquinazolines (2a-c), as illustrated in Scheme 2. The second route began with substituted 2-thioxoquinazolin-4(1*H*)-ones (3a-c) and terminated with the benzylation of quinazolinethione, as detailed in Scheme 3.

**SCHEME 1:**
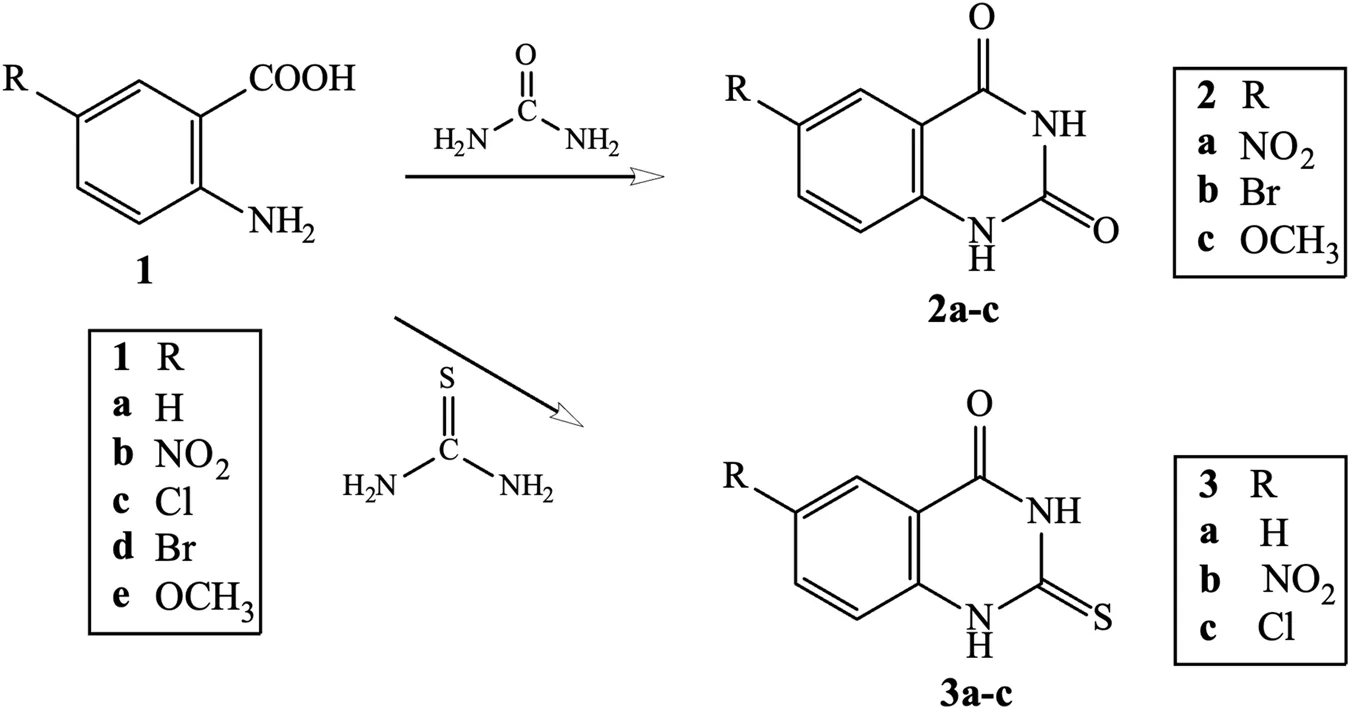
Synthesis of quinazolone derivatives.

**SCHEME 2:**
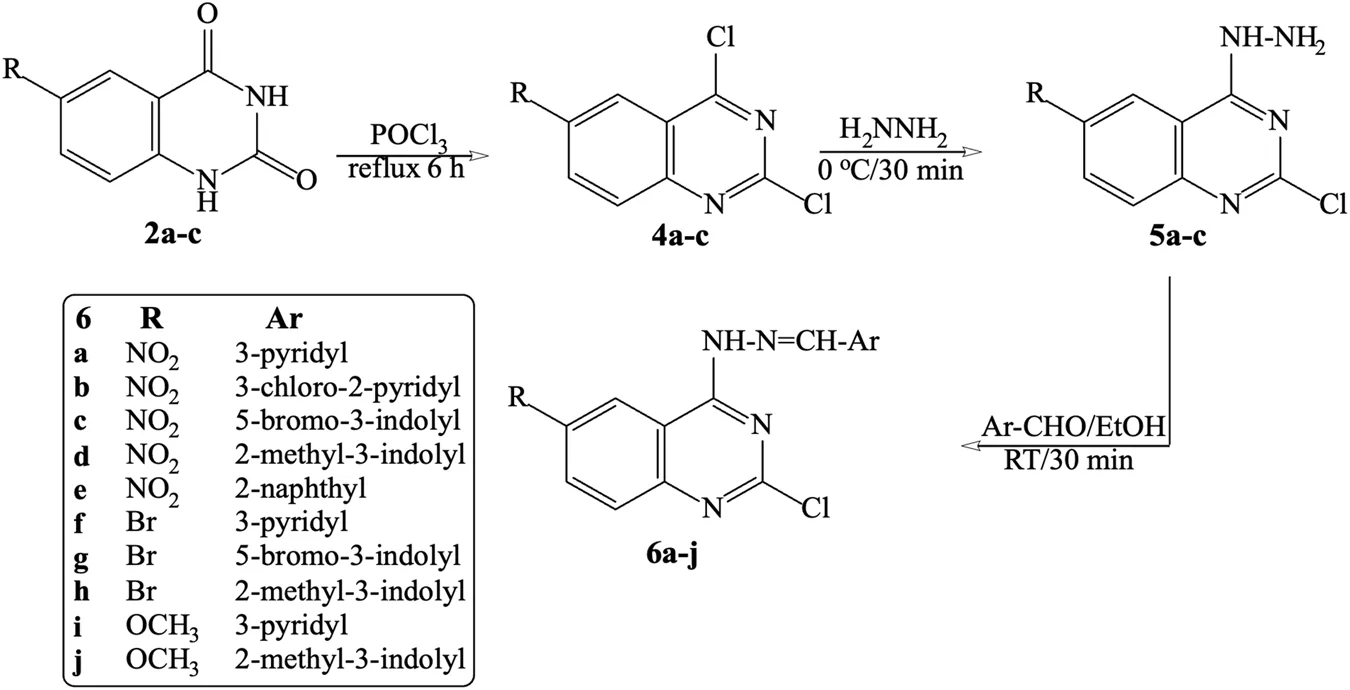
Preparation of hydrazone derivatives.

Initially, the quinazolone nuclei (2a-c and 3a-c) were synthesized according to reported procedures (Russo and Ghelardoni, 1967) *via* cyclization of substituted anthranilic acid (1) with urea or thiourea, respectively (Scheme 1). The hydrazone derivatives were then synthesized following the reaction sequence outlined in Scheme 2. Briefly, 2,4-dichloroquinazolines (4a-c) were synthesized by chlorination of 2,4-dioxoquinazolines (2a-c) using a mixture of phosphoryl chloride and *N*,*N*-dimethylaniline. This method follows established procedures and effectively introduces chlorine substituents at the 2 and 4 positions of the quinazoline ring (Curd et al., 1947; El-Kerdawy et al., 1990). Subsequently, the 2,4-dichloroquinazolines (4a-c) react with anhydrous hydrazine through preferential monohydrazinolysis, leading to the formation of hydrazinoquinazolines (5a-c). The increased reactivity at the C4 position, attributed to the chlorine substituents, promotes this selective transformation, resulting in the desired hydrazino derivatives.

Finally, Schiff bases were synthesized through the classic condensation reaction between hydrazinoquinazolines (5a-c) and a carbonyl compound, specifically an aldehyde, using ethanol as a solvent. This reaction resulted in the formation of 4-hydrazonoquinazolines (6a-j). The structures of the synthesized 4-hydrazonoquinazoline derivatives (6a-j) were confirmed using a combination of analytical tools, including ^1^HNMR, ^13^CNMR spectroscopy, mass spectrometry, infrared spectroscopy, and elemental analysis. The IR spectra showed absorption bands corresponding to the NH group in the range of 3356–3182 cm^-1^. Additionally, the ^1^HNMR spectra of the arylidenehydrazine derivatives (6a-j) exhibited signals for the ArCH = N moiety at 8.27–6.90 ppm. Furthermore, the NH proton of the hydrazone moiety exists in tautomeric equilibrium with the adjacent ring nitrogen atom and appears as a doublet signal in the range of δ 11.80–11.29 ppm. In addition, the NH proton of the indole ring is observed as a singlet signal in the range of δ 9.93–11.98 ppm. Furthermore, compounds 7 and 8 contain another NH functionality that is involved in tautomerism with the corresponding amide carbonyl oxygen. This NH proton resonates at δ 6.01–4.81 ppm.; however, E/Z isomerism was not experimentally assigned in the current study.

**SCHEME 3:**
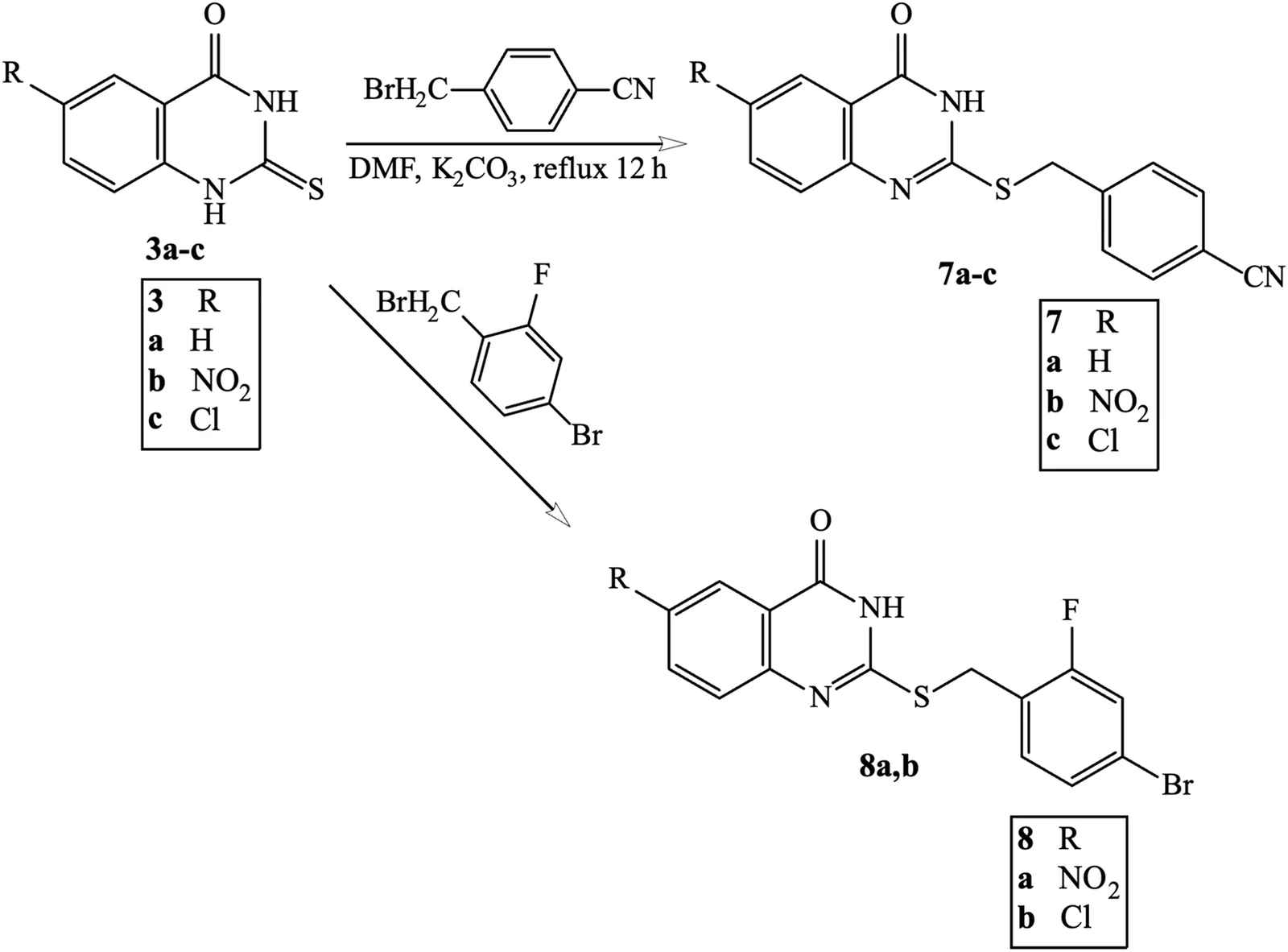
Benzylation of quinazolinethione derivatives.

The second synthetic route involved the benzylation of quinazolinethione derivatives, following the reaction sequence presented in Scheme 3. *S*-alkylation of quinazolinethione derivatives was performed according to established procedures (Sánchez et al., 2013), utilizing various benzyl bromides in DMF (*N*,*N*-dimethylformamide) and in the presence of potassium carbonate. The reaction was conducted under reflux conditions for 12 h, yielding compounds 7a-c and 8a,b. The structures of compounds 7a-c and 8a,b were characterized by IR,^1^HNMR, ^13^CNMR spectroscopy, mass spectrometry, and elemental analysis. In the IR spectra, absorption bands corresponding to the NH group appeared in the range of 3394–3155 cm^-1^, while the CN group was observed at 2225 cm^-1^. The ^1^HNMR spectra exhibited a singlet signal at 2.97–2.73 ppm, corresponding to the CH_2_ protons, as well as an exchangeable OH signal with D_2_O at δ 6.01–4.81 ppm.

### 3.2 Acute oral toxicity and *in vivo* anti-inflammatory activity

The synthesized quinazoline derivatives were initially subjected to acute oral toxicity in mice. The highest tested doses not associated with mortality or overt clinical toxicity ranged from 1000 to 1200 mg/kg under the study conditions. Because no formal statistical LD_50_ determination was performed, these values were considered preliminary oral tolerability estimates. One-tenth of the corresponding highest non-toxic tested dose was subsequently selected for anti-inflammatory evaluation, resulting in test doses of 100 or 120 mg/kg for the synthesized compounds (Table 1).

**TABLE 1:** Preliminary acute oral tolerability estimates and selected doses for anti-inflammatory evaluation.

Compound	Highest tested oral dose without observed mortality or overt clinical toxicity (mg/kg)	Selected anti-inflammatory test dose (mg/kg)
6a	1200	120
6b	1200	120
6c	1000	100
6d	1200	120
6e	1200	120
6f	1000	100
6g	1000	100
6h	1200	120
6i	1200	120
6j	1000	100
7a	1000	100
7b	1000	100
7c	1000	100
8a	1200	120
8b	1000	100

The anti-inflammatory test dose was selected as one-tenth of the corresponding highest tested dose without observed mortality or overt clinical toxicity. Diclofenac was administered at 7.5 mg/kg.

**FIGURE 1:**
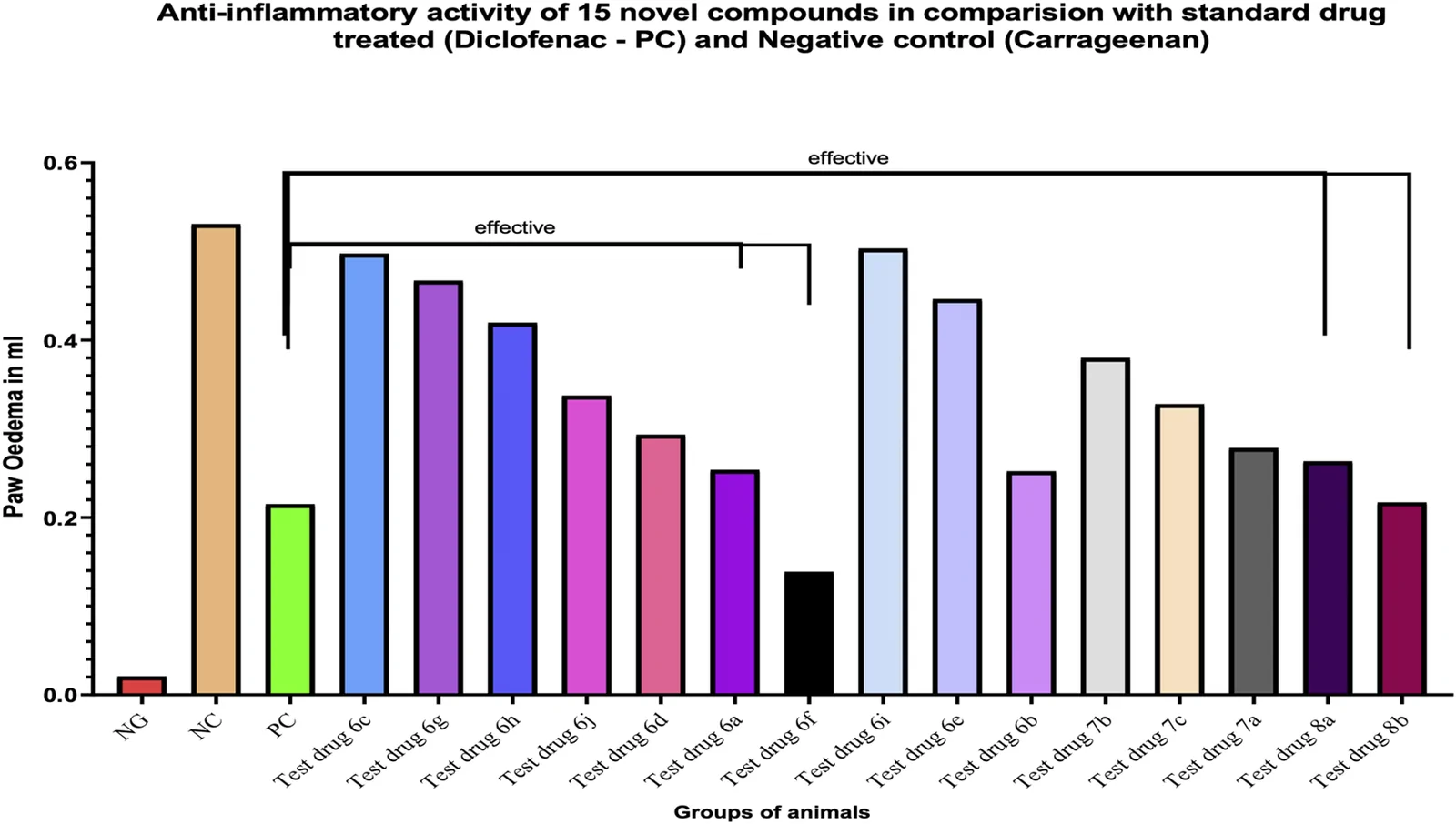
Comparative percentage inhibition of carrageenan-induced paw edema at 3 h for diclofenac and the four most active quinazoline derivatives (6a, 6b, 6f, 8a). Data represent mean ± SEM (n = 6). Statistical significance was determined by one-way ANOVA with Dunnett’s test.

Anti-inflammatory activity was evaluated using the carrageenan-induced rat paw-edema model. Paw volume was measured at baseline and at 1, 2, and 3 h after carrageenan administration. At 3 h, the mean paw-edema increase in the disease-control group was 0.865 mL. Diclofenac administered at 7.5 mg/kg reduced the mean paw-edema increase to 0.275 mL, corresponding to 68.21% inhibition (Table 2; Figure 1).

**TABLE 2:** Effect of the synthesized quinazoline derivatives on carrageenan-induced paw edema at 3 h.

Group	Mean increase (3h, mL)	% inhibition	Conceptual category
Normal group	0.01833 ± 0.003073	-	-
Disease control	0.865 ± 0.01232	References	References
Diclofenac	0.275 ± 0.007638	68.21	Standard drug
6c	0.8333 ± 0.01978###	3.66	Inactive
6g	0.7867 ± 0.006667**###	9.05	Inactive
6h	0.73 ± 0.01183***###	15.61	Weakly active
6j	0.5433 ± 0.008433***###	37.19	Moderately active
6d	0.465 ± 0.01176***###	46.24	Moderately active
6a	0.3300 ± 0.01125***##	61.85	Highly active
6f	0.1783 ± 0.004014***##	79.39	Very highly active
6i	0.84 ± 0.005774###	2.89	Inactive
6e	0.7517 ± 0.006009***###	13.1	Weakly active
6b	0.3317 ± 0.01078***##	61.65	Highly active
7b	0.6417 ± 0.006009***###	25.82	Weakly active
7c	0.5617 ± 0.01302***###	35.06	Moderately active
7a	0.4783 ± 0.006009***###	44.71	Moderately active
8a	0.3450 ± 0.01232***###	60.12	Highly active
8b	0.4233 ± 0.04499***###	51.06	Moderately active

Values are presented as mean ± SEM (n = 6). Statistical analysis was performed using one-way ANOVA, followed by Dunnett’s multiple-comparisons test in GraphPad Prism version 9.5.1. Asterisks indicate statistical significance versus the disease-control group, whereas hash symbols indicate statistical significance versus the diclofenac-treated group: *p ≤ 0.05, **p ≤ 0.01, and ***p ≤ 0.001; #p ≤ 0.05, ##p ≤ 0.01, and ###p ≤ 0.001.

Among the tested compounds, 6f showed the highest numerical inhibition of paw edema, with 79.39% inhibition at 100 mg/kg. Compounds 6a, 6b, and 8a, each evaluated at 120 mg/kg, produced inhibition values of 61.85%, 61.65%, and 60.12%, respectively. Because the synthesized compounds and diclofenac were evaluated at different doses and no dose–response analysis was performed, these findings represent the observed effects under the study conditions and should not be interpreted as direct evidence of comparative potency.

For descriptive interpretation, compounds were categorized according to their percentage inhibition at 3 h as follows: <10% (inactive), 10%–34% (weakly active), 35%–59% (moderately active), 60%–74% (highly active), and ≥75% (very highly active).

### 3.3 Molecular modeling

#### 3.3.1 Molecular docking study

Molecular docking was performed to evaluate the predicted binding poses and relative computational scores of selected biologically active quinazoline derivatives within the PDE7A catalytic site. The Induced Fit Docking (IFD) workflow was employed to account for local receptor flexibility during ligand placement and refinement. Glide GScore and IFDScore were used to rank the predicted docking poses, whereas Prime MM–GBSA ΔG_bind was applied as a complementary post-docking estimate of binding energetics. More negative values within each individual scoring method were interpreted as more favorable; however, the different metrics were not considered directly interchangeable or definitive measures of experimental binding affinity.

The co-crystallized thioxoquinazolinone inhibitor TC8 from the PDE7A crystal structure (PDB ID: 3G3N) was used as a reference ligand (Castaño et al., 2009). Re-docking of TC8 reproduced its crystallographic pose with an RMSD of 1.69 Å, confirming the reliability of the docking protocol.

The four selected compounds, 6f, 6b, 6a, and 8a, exhibited more favorable Glide GScores than TC8, which had a Glide GScore of −5.897 (Table 3). However, the Prime MM–GBSA estimates did not follow the same ranking, demonstrating that the docking and rescoring results should be interpreted as complementary computational metrics.

**TABLE 3:** Induced-fit docking and post-docking computational scores of selected biologically active compounds and the co-crystallized reference ligand TC8 within the PDE7A catalytic site.

Compound	Docking score	Glide emodel	Glide gscore	IFDScore	Prime energy	MM–GBSA ΔG_bind (kcal/mol)
6f	−9.229	−73.712	−9.241	−727.94	−14373.88	−40.92
6 b	−8.678	−76.389	−8.714	−725.25	−14193.85	−53.48
6a	−8.522	−73.240	−8.558	−726.83	−14228.51	−56.12
8a	−6.519	−70.534	−7.532	−720.93	−14134.07	−61.36
TC8-3G3N	−5.897	−46.779	−5.897	−718.15	−14105.25	−57.08

Docking results of the selected biologically active compounds with PDE7A.

**FIGURE 2:**
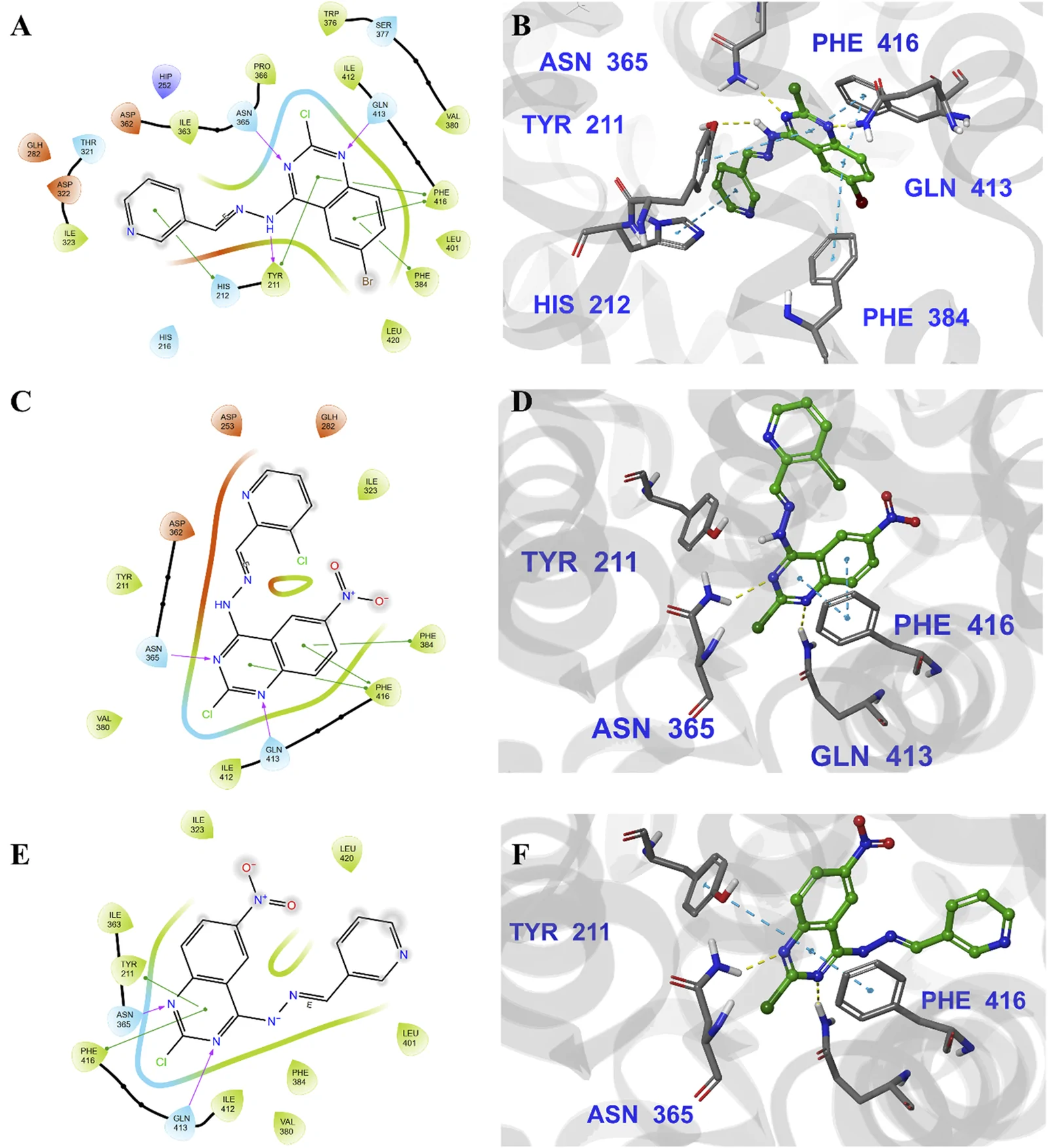
Representative 2D and 3D interaction maps of selected biologically active compounds within the PDE7A active site **(A)** 2D interaction map of 6f **(B)** 3D interaction map of 6f **(C)** 2D interaction map of 6b **(D)** 3D interaction map of 6b **(E)** 2D interaction map of 6a **(F)** 3D interaction map of 6a.

Compound 6f was the highest-ranked compound according to Glide GScore and IFDScore, with values of −9.241 and −727.94, respectively. Its Prime MM–GBSA ΔG_bind value was −40.92 kcal/mol, which was less favorable than that of TC8 (−57.08 kcal/mol). In the predicted docking pose, 6f formed hydrogen bonds with Asn365, Gln413, and Tyr211; π–π interactions with Phe384, Phe416, and His212; and hydrophobic contacts involving Leu401, Leu420, Val380, Ile412, Ile363, and Trp376 (Figures 2A,B).

Compounds 6b and 6a also showed favorable Glide GScores of −8.714 and −8.558, respectively. Their Prime MM–GBSA ΔG_bind values were −53.48 and −56.12 kcal/mol, respectively, and were close to the corresponding estimate for TC8. Their predicted docked poses included hydrogen-bonding interactions with Asn365 and Gln413, aromatic interactions involving Phe416, Phe384, and Tyr211, and hydrophobic contacts with Leu401, Val380, Ile412, Trp376, and Leu420 (Figures 2C–F).

Compound 8a demonstrated a Glide GScore of −7.532 and the most negative MM–GBSA ΔG_bind value among the evaluated compounds (−61.36 kcal/mol). The discrepancy between its comparatively weaker Glide ranking and more favorable MM–GBSA estimate illustrates the limitations of prioritizing compounds using a single computational score. Accordingly, the docking and MM–GBSA findings were considered together with the subsequent molecular dynamics analyses rather than being interpreted independently as evidence of binding affinity or stability.

#### 3.3.2 Molecular dynamics simulations

Compounds 6f, 6a, 6b, and 8a were further evaluated using 100-ns MD simulations to examine the comparative dynamic behavior of their predicted PDE7A complexes relative to the co-crystallized reference ligand TC8. Protein conformational behavior was monitored using protein Cα RMSD and residue-level Cα RMSF, whereas ligand positional behavior was assessed using ligand RMSD relative to the aligned protein. Residue-specific protein–ligand contacts were evaluated using quantitative interaction occupancies and timelines. Trajectory behavior was further examined using block-wise analysis of five sequential 20-ns intervals.

**FIGURE 3:**
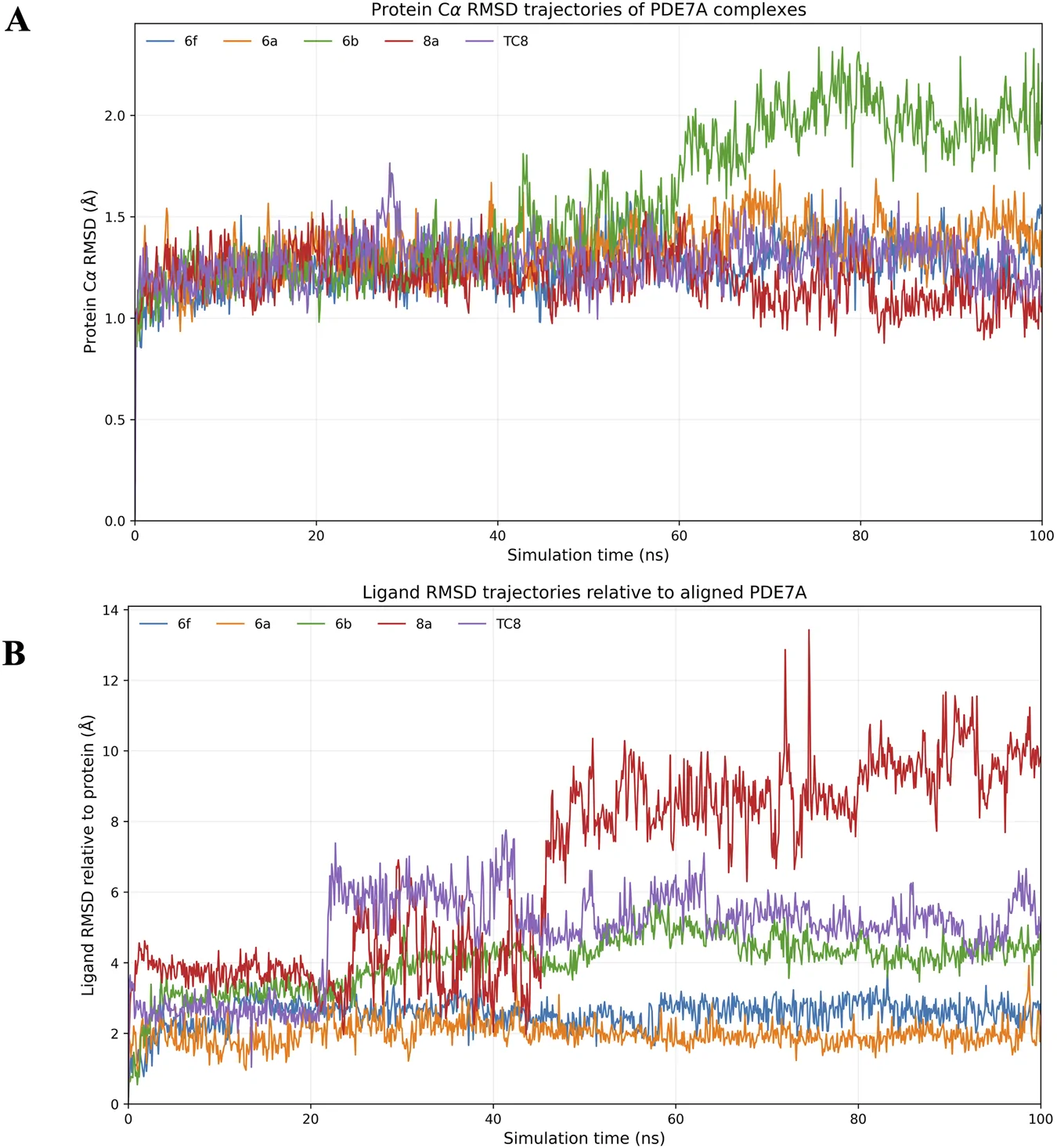
Comparative RMSD trajectories of the simulated PDE7A complexes over 100 ns. **(A)** Protein Cα RMSD trajectories for PDE7A complexes with compounds 6f, 6a, 6b, and 8a and the co-crystallized reference ligand TC8. **(B)** Ligand RMSD trajectories calculated relative to the aligned PDE7A protein.

For the PDE7A–6f complex, the protein Cα RMSD reached an apparent late-stage stationary regime, with mean values of 1.298 ± 0.099 Å and 1.299 ± 0.106 Å during 60–80 and 80–100 ns, respectively. The corresponding ligand RMSD values were also similar during these intervals, at 2.689 ± 0.295 Å and 2.661 ± 0.311 Å (Figure 3; Supplementary Table S1). Quantitative interaction analysis showed highly persistent hydrogen bonds with Tyr211 and Gln413, present in 96.70% and 92.21% of the analyzed frames, respectively. Persistent π–π interactions were also observed with Phe416 and Phe384, with occupancies of 93.21% and 62.34%, respectively. Additional contacts included hydrogen bonding with Lys359 and hydrophobic interactions involving Ile363, Val380, Ile412, Ile323, and Leu420 (Figure 3; Supplementary Tables S1, S2; Supplementary Figure S1).

The PDE7A–6a complex also approached an apparent stationary RMSD regime during the final 40 ns? Protein Cα RMSD values were 1.437 ± 0.110 Å and 1.401 ± 0.096 Å during the final two intervals, while the corresponding ligand RMSD values were 1.894 ± 0.237 Å and 1.960 ± 0.327 Å. Its principal interactions included π–π stacking with Phe416 and hydrogen bonding with Gln413, observed in 99.10% and 95.90% of the analyzed frames, respectively. Hydrogen bonding with Tyr211 and π–π interaction with Phe384 were also persistent, with occupancies of 65.23% and 67.13%, respectively (Figure 3; Supplementary Tables S1, S2; Supplementary Figure S2).

In contrast, the PDE7A–6 b complex underwent a substantial conformational adjustment during the middle portion of the trajectory before approaching a late quasi-stationary state. Its protein Cα RMSD increased to 1.957 ± 0.156 Å and 1.975 ± 0.121 Å during 60–80 and 80–100 ns, respectively, while the corresponding ligand RMSD values were 4.583 ± 0.364 Å and 4.286 ± 0.304 Å. The dominant interaction was π–π stacking with Phe416, present in 87.11% of frames, followed by hydrogen bonding with Gln413 at 56.64% and π–π interaction with Phe384 at 49.05%. Persistent hydrophobic contacts were also observed with Val380, Leu420, Ile323, and Ile412 (Figure 3; Supplementary Tables S1, S2; Supplementary Figure S3).

For the PDE7A–8a complex, the protein Cα RMSD remained comparatively restrained, whereas the ligand RMSD showed a marked transition after approximately 45–50 ns. The mean ligand RMSD increased to 8.572 ± 0.904 Å during 60–80 ns and 9.726 ± 0.694 Å during 80–100 ns, indicating substantial displacement from the initial docked pose and continued positional adjustment during the latter part of the trajectory. The principal retained interaction was π–π stacking with Phe416, observed in 62.14% of frames. Hydrogen bonds with Asn327, Asp362, and Ile323 showed occupancies of 25.77%, 18.98%, and 12.99%, respectively, while the main hydrophobic contacts involved Leu401, Leu420, Val380, and Ile323. The trajectory was therefore not considered converged to the initial docking pose (Figure 3; Supplementary Tables S1, S2; Supplementary Figure S4).

The PDE7A–TC8 complex maintained a comparatively stable protein backbone but underwent an early ligand-pose transition before approaching a different late-stage configuration. Protein Cα RMSD values were 1.310 ± 0.092 Å and 1.286 ± 0.103 Å during the final two intervals, while ligand RMSD values were 5.368 ± 0.481 Å and 5.120 ± 0.510 Å. The dominant interaction was π–π stacking with Phe384, present in 68.03% of frames. Additional contacts included a water bridge with Pro400 at 39.16%, hydrogen bonding with Asn260 at 28.67%, and less persistent aromatic, hydrophobic, and water-mediated interactions (Figure 3; Supplementary Tables S1, S2; Supplementary Figure S5).

**FIGURE 4:**
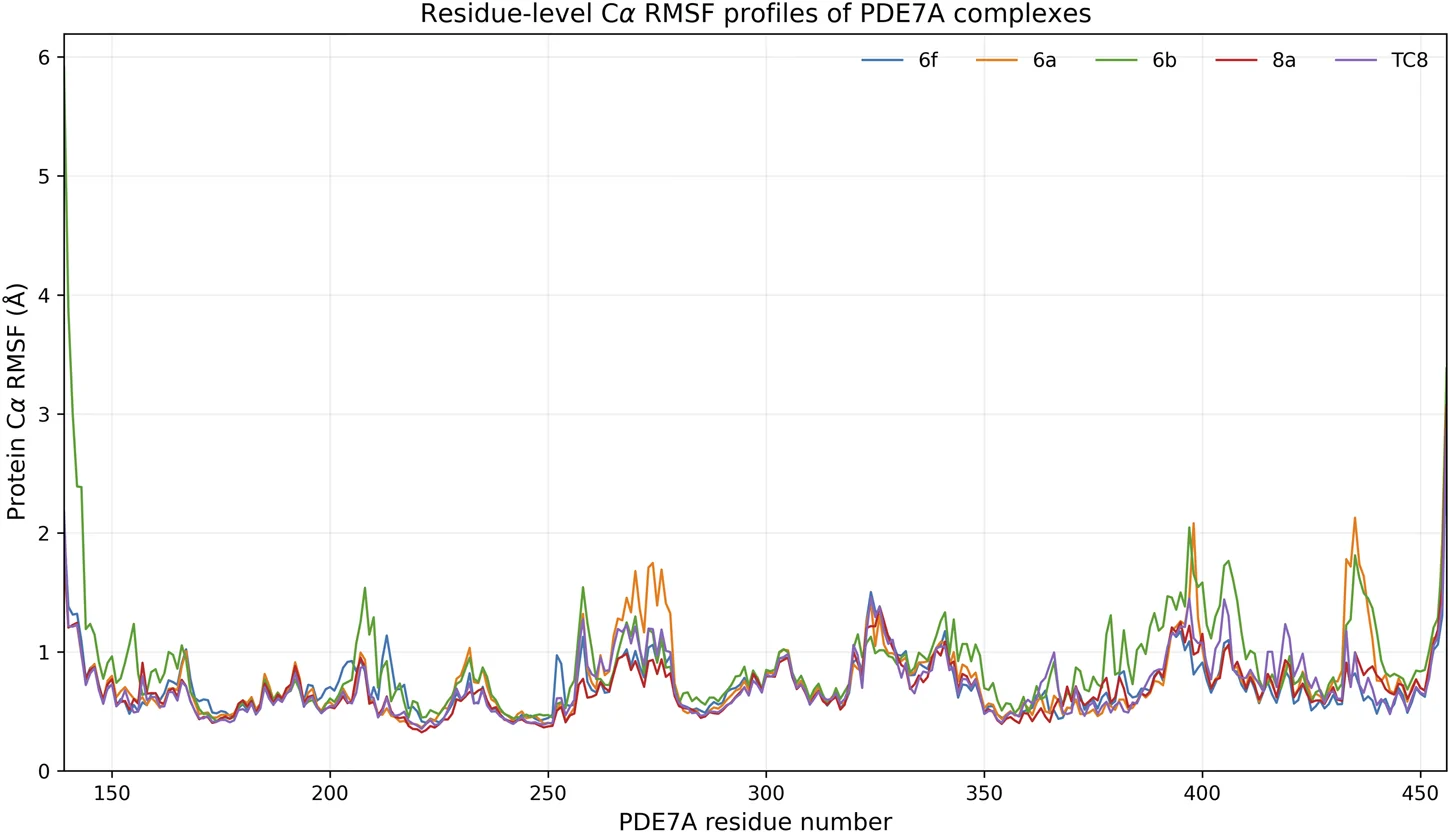
Comparative protein Cα RMSF profiles of the simulated PDE7A complexes. Residue-level Cα RMSF profiles are shown for PDE7A complexes with compounds 6f, 6a, 6b, and 8a and the co-crystallized reference ligand TC8. Identical residue numbering and axis scales were used for all systems.

Protein Cα RMSF analysis showed generally low-to-moderate residue-level fluctuations across the five systems. Mean Cα RMSF values were 0.696, 0.720, 0.721, 0.770, and 0.897 Å for the 8a, 6f, TC8, 6a, and 6 b complexes, respectively. The largest fluctuations were mainly confined to terminal and flexible loop regions. Most binding-site residues, including Tyr211, Gln413, Phe384, and Phe416, remained comparatively restrained. The 6 b complex showed the greatest overall protein flexibility and moderately elevated fluctuations around residues 397–402, consistent with the conformational adjustment observed in its RMSD trajectory (Figure 4).

Considering the biological and computational findings together, compound 6f showed the most consistent overall profile among the evaluated derivatives and was selected as the leading candidate for further investigation. This prioritization does not imply confirmed PDE7A engagement or long-term binding stability.

### 3.4 In silico ADMET analysis

The predicted ADMET properties of the selected biologically active compounds 6f, 6a, 6b, and 8a were evaluated using the QikProp module to characterize their drug-likeness, absorption-related properties, distribution profile, and potential safety liabilities (Table 4).

**TABLE 4:** In silico ADMET prediction for the selected biologically active compounds.

Descriptor	Recommended values	6f	6 b	6a	8a
#Stars	0–5	0	1	1	1
#rtvFG	0–2	1	1	1	0
CNS	−2 (inactive), +2 (active)	0	−2	−2	−2
mol_MW	130.0–725.0	362.616	363.162	328.717	410.217
SASA	300.0–1000.0	573.475	606.457	583.569	608.518
Volume	500.0–2000.0	951.894	1012.534	972.413	1022.166
donorHB	0.0–6.0	1	1	1	1
accptHB	2.0–20.0	6	6.5	7	4.5
QPlogPw	4.0–45.0	10.398	11.04	11.752	9.199
QPlogPo/w	−2.0 – 6.5	3.046	2.641	1.91	3.38
QPlogS	−6.5 – 0.5	−4.471	−4.484	−3.96	−5.724
QPlogHERG	Concern below −5	−6.067	−6.048	−6.06	−5.852
QPPCaco	<25 poor, >500 great	1807.933	263.985	214.926	176.445
QPlogBB	−3.0 – 1.2	−0.081	−1.105	−1.318	−1.206
#Metab	1–8	2	2	3	2
QPlogKhsa	−1.5 – 1.5	−0.124	−0.114	−0.308	0.348
Human oral absorption	1, 2, or 3 for low, medium, or high	3	3	3	3
Percent human oral absorption	>80% is high; <25% is poor	100	85.753	79.872	86.943

All four compounds demonstrated zero violations of Lipinski’s rule of five and low numbers of property outliers (#stars = 0–1). Their molecular weights ranged from 328.7 to 410.2 Da, while the predicted hydrogen-bond donor and acceptor counts were 1 and 4.5–7, respectively, remaining within commonly accepted drug-like ranges.

The predicted QPlogP_o/w values ranged from 1.91 to 3.38, indicating moderate lipophilicity. Predicted aqueous solubility values ranged from −3.96 to −5.72, suggesting moderate-to-low solubility across the series, with compound 8a showing the least favorable predicted solubility among the evaluated compounds.

All compounds exhibited high predicted human oral absorption score of 3, corresponding to high predicted absorption, with estimated percentage absorption values ranging from 79.9% to 100%. Predicted Caco-2 permeability values ranged from 176 to 1807 nm/s, indicating moderate-to-high intestinal permeability, with compound 6f showing the highest predicted permeability.

The predicted blood–brain barrier partition coefficients (QPlogBB) ranged from −1.318 to −0.08, indicating variable but generally limited-to-moderate predicted CNS penetration. Reduced CNS exposure may be advantageous for a peripherally acting anti-inflammatory agent because it could limit unnecessary central exposure and potential CNS-related adverse effects. Conversely, restricted blood–brain barrier penetration would be disadvantageous for neuroinflammatory indications requiring direct CNS activity. The relevance of this profile therefore depends on the intended therapeutic application and requires experimental confirmation.

The predicted QPlogHERG values ranged from −5.85 to −6.07, falling below the QikProp concern threshold of −5.0. These values represent an *in silico* safety alert for possible hERG channel inhibition and should not be interpreted as experimental evidence of cardiotoxicity. Direct hERG evaluation should therefore be prioritized during subsequent lead optimization, together with structural modifications aimed at reducing potential channel liability.

Overall, the evaluated compounds showed acceptable predicted drug-likeness and absorption-related properties, but the predicted aqueous solubility and hERG liability require further investigation. Compound 6f displayed a comparatively balanced predicted profile and produced the highest numerical inhibition of paw edema at its tested dose; however, experimental pharmacokinetic and safety studies are required before its developability can be established.

## 4 Discussion

The present study identified several quinazoline derivatives with notable anti-inflammatory activity and used structure-based computational analyses to explore their possible interaction with PDE7A. Among the tested compounds, 6f showed the strongest numerical inhibition of paw edema at its administered dose and was therefore prioritized for further evaluation. Compounds 6a, 6b, and 8a also showed substantial activity, supporting the potential of both the hydrazone and benzylthioquinazolinone series as sources of anti-inflammatory leads.

The computational results supported the prioritization of 6f. It achieved the most favorable Glide GScore and IFDScore among the evaluated compounds and maintained persistent predicted interactions with residues within the PDE7A catalytic site, particularly Tyr211, Gln413, Phe384, and Phe416. Its molecular dynamics trajectory also showed comparatively consistent late-stage behavior. By contrast, the different ranking obtained from MM–GBSA, particularly the favorable estimate for 8a, highlights the value of evaluating docking, rescoring, interaction persistence, and trajectory behavior together rather than relying on a single computational parameter.

The molecular dynamics simulations further distinguished the selected compounds. Compound 6a retained several persistent polar and aromatic contacts and showed relatively consistent behavior during the final portion of the trajectory. Compound 6 b underwent a conformational adjustment before approaching a late quasi-stationary configuration, whereas 8a showed greater displacement from its initial docked pose. The co-crystallized ligand TC8 also adopted a different late-stage orientation during the simulation. Overall, 6f showed the most balanced dynamic and interaction profile among the evaluated compounds.

The biological data allowed a preliminary descriptive SAR analysis. Compounds 6f, 6a, and 6i share the same 2-chloro-4-(3-pyridylmethylenehydrazino) framework but differ in their C6 substituents, bearing bromo, nitro, and methoxy groups, respectively. Their observed inhibition values followed the order 6f (79.39%) > 6a (61.85%) >> 6i (2.89%). Although the single-dose design prevents direct potency comparisons, this pattern suggests that the physicochemical nature of the C6 substituent influenced the biological response. The bromo substituent in 6f may provide a favorable combination of hydrophobicity, polarizability, and steric compatibility than the more strongly polar nitro substituent in 6a or the electron-donating methoxy group in 6i.

Compound 6 b contains an additional chlorine substituent on the pyridyl ring and showed an inhibition value similar to that of 6a at the same tested dose. This observation suggests that further halogenation of the heteroaromatic ring was tolerated but did not provide a clear activity advantage. The apparent contribution of electron-withdrawing groups and pyridyl substitution is broadly consistent with previous reports on related quinazoline derivatives, in which these structural features were associated with favorable physicochemical or predicted protein-binding properties (El-Malah et al., 2024). Nevertheless, this comparison should be regarded as qualitative because differences in scaffold, assay conditions, and biological targets may limit direct extrapolation.

Replacement of the relatively compact pyridyl hydrazone moiety with bulkier indolyl or naphthyl substituents was generally associated with lower observed activity. This may reflect increased steric demand, altered conformational preferences, reduced solubility, or less favorable accommodation within the relevant biological binding environment. Taken together, the biological and computational findings suggest that the favorable profile of 6f may arise from the combined contribution of its bromo-substituted quinazoline core, pyridyl hydrazone moiety, and predicted interactions within the PDE7A catalytic site.

The *in silico* ADMET analysis indicated that the selected compounds satisfied Lipinski’s rule-of-five criteria and showed generally favorable predicted absorption-related properties. Compound 6f exhibited particularly high predicted Caco-2 permeability and oral absorption. However, the moderate-to-low predicted aqueous solubility of the series and the QPlogHERG values of −5.85 to −6.07 represent potential development concerns. The predicted hERG values fall below the QikProp concern threshold of −5.0 and should therefore be regarded as an *in silico* safety signal rather than evidence of experimentally confirmed channel inhibition or cardiotoxicity. Experimental solubility, pharmacokinetic, and hERG studies will be needed to assess the developability of the prioritized compounds.

Overall, compound 6f showed the most favorable combined biological and computational profile within the evaluated series. These findings support its selection for further investigation, while direct biochemical and pharmacological studies are required to clarify the contribution of PDE7A to its anti-inflammatory activity.

## 5 Limitations

Anti-inflammatory activity was evaluated at single, non-equivalent doses, preventing dose–response analysis and direct comparison of potency. PDE7A inhibition, target engagement, and selectivity against related phosphodiesterases were not experimentally assessed. The molecular dynamics analysis was limited to single 100-ns trajectories, and the predicted ADMET properties, including potential hERG liability, require experimental confirmation. The animal study was also not designed to evaluate sex-specific effects.

## Data Availability

The original contributions presented in the study are included in the article/Supplementary Material, further inquiries can be directed to the corresponding author.
