# Supplementary material for: New quinazoline derivatives with anti-inflammatory activity and computationally predicted PDE7A binding: synthesis, molecular modeling, and in silico pharmacokinetic evaluation
Source: Front Pharmacol. 2026 Aug 21;17:1859798. doi: 10.3389/fphar.2026.1859798 (PMC13541538; doi:10.3389/fphar.2026.1859798)
Supplement: Supplementary file 1 [file Table1.pdf]

Supplementary Table S1. Block-wise RMSD statistics for the simulated PDE7A complexes during the 100-ns molecular dynamics simulations.

Complex	RMSD measure	0–20 ns	20–40 ns	40–60 ns	60–80 ns	80–100 ns
6f	Protein Cα RMSD	1.155 ± 0.137	1.257 ± 0.096	1.286 ± 0.121	1.298 ± 0.099	1.299 ± 0.106
	Ligand RMSD relative to aligned protein	2.326 ± 0.600	2.707 ± 0.262	2.427 ± 0.298	2.689 ± 0.295	2.661 ± 0.311
6a	Protein Cα RMSD	1.211 ± 0.153	1.301 ± 0.100	1.355 ± 0.092	1.437 ± 0.110	1.401 ± 0.096
	Ligand RMSD relative to aligned protein	1.815 ± 0.398	2.223 ± 0.341	2.050 ± 0.287	1.894 ± 0.237	1.960 ± 0.327
6b	Protein Cα RMSD	1.208 ± 0.132	1.260 ± 0.092	1.467 ± 0.129	1.957 ± 0.156	1.975 ± 0.121
	Ligand RMSD relative to aligned protein	2.912 ± 0.678	3.817 ± 0.458	4.476 ± 0.488	4.583 ± 0.364	4.286 ± 0.304
8a	Protein Cα RMSD	1.222 ± 0.141	1.253 ± 0.098	1.216 ± 0.096	1.181 ± 0.120	1.087 ± 0.098
	Ligand RMSD relative to aligned protein	3.748 ± 0.428	4.019 ± 1.060	7.086 ± 2.153	8.572 ± 0.904	9.726 ± 0.694
TC8	Protein Cα RMSD	1.200 ± 0.121	1.354 ± 0.115	1.270 ± 0.095	1.310 ± 0.092	1.286 ± 0.103
	Ligand RMSD relative to aligned protein	2.640 ± 0.352	5.580 ± 1.037	5.392 ± 0.745	5.368 ± 0.481	5.120 ± 0.510

Note. Values are presented as mean ± SD in Å. Protein RMSD was calculated using Cα atoms, and ligand RMSD was calculated relative to the aligned PDE7A protein. The trajectory was divided into five sequential 20-ns intervals; the 100-ns endpoint was included in the final interval.
