# Supplementary material for: New quinazoline derivatives with anti-inflammatory activity and computationally predicted PDE7A binding: synthesis, molecular modeling, and in silico pharmacokinetic evaluation
Source: Front Pharmacol. 2026 Aug 21;17:1859798. doi: 10.3389/fphar.2026.1859798 (PMC13541538; doi:10.3389/fphar.2026.1859798)
Supplement: Supplementary file 2 [file Table2.pdf]

Table S2. Residue-specific protein–ligand interaction occupancies during 100-ns molecular dynamics simulations of PDE7A complexes.

Complex	Residue	Interaction type	Occupancy (%)
6f	Tyr211	Hydrogen bond	96.70
6f	Gln413	Hydrogen bond	92.21
6f	Lys359	Hydrogen bond	37.96
6f	Asn365	Hydrogen bond	2.70
6f	His256	Hydrogen bond	0.90
6f	Gln326	Hydrogen bond	0.10
6f	Phe416	π–π	93.21
6f	Phe384	π–π	62.34
6f	His212	π–π	23.88
6f	Tyr211	π–π	4.30
6f	His256	π–π	0.60
6f	Lys359	π–cation	2.40
6f	Ile363	Hydrophobic	27.67
6f	Val380	Hydrophobic	21.48
6f	Ile412	Hydrophobic	15.38
6f	Ile323	Hydrophobic	12.79
6f	Leu420	Hydrophobic	12.79
6f	Phe384	Hydrophobic	7.09
6f	Phe416	Hydrophobic	5.79
6f	Leu401	Hydrophobic	1.30
6a	Gln413	Hydrogen bond	95.90
6a	Tyr211	Hydrogen bond	65.23
6a	Asn260	Hydrogen bond	1.00
6a	Asn365	Hydrogen bond	0.20
6a	Phe416	π–π	99.10
6a	Phe384	π–π	67.13
6a	His212	π–π	34.07
6a	Tyr211	π–π	33.87
6a	Phe416	π–cation	4.50
6a	Lys359	π–cation	1.70
6a	Phe384	π–cation	0.30
6a	His252	π–cation	0.10
6a	Val380	Hydrophobic	31.37
6a	Ile323	Hydrophobic	24.08
6a	Ile412	Hydrophobic	8.99
6a	Pro366	Hydrophobic	3.30
6a	Phe384	Hydrophobic	2.80
6a	Leu401	Hydrophobic	1.20
6a	Tyr211	Hydrophobic	0.80
6a	Phe416	Hydrophobic	0.70
6b	Gln413	Hydrogen bond	56.64
6b	Tyr211	Hydrogen bond	22.38
6b	Cys402	Hydrogen bond	4.70
6b	Asp362	Hydrogen bond	2.50
6b	Ser377	Hydrogen bond	0.60
6b	Asn365	Hydrogen bond	0.20
6b	Phe416	π–π	87.11
6b	Phe384	π–π	49.05
6b	Tyr211	π–π	22.48
6b	Phe384	π–cation	27.27

Complex	Residue	Interaction type	Occupancy (%)
6b	Phe416	π–cation	17.38
6b	Lys359	π–cation	0.50
6b	Val380	Hydrophobic	39.36
6b	Leu420	Hydrophobic	35.16
6b	Ile323	Hydrophobic	31.57
6b	Ile412	Hydrophobic	23.28
6b	Ile363	Hydrophobic	9.69
6b	Pro366	Hydrophobic	5.59
6b	Phe384	Hydrophobic	5.00
6b	Phe416	Hydrophobic	4.50
6b	Leu424	Hydrophobic	0.90
6b	Leu401	Hydrophobic	0.40
6b	Tyr211	Hydrophobic	0.10
8a	Asn327	Hydrogen bond	25.77
8a	Asp362	Hydrogen bond	18.98
8a	Ile323	Hydrogen bond	12.99
8a	Gln326	Hydrogen bond	7.29
8a	Lys359	Hydrogen bond	1.40
8a	Gln261	Hydrogen bond	1.00
8a	Glu328	Hydrogen bond	0.60
8a	His256	Hydrogen bond	0.50
8a	Asn260	Hydrogen bond	0.30
8a	Ser324	Hydrogen bond	0.10
8a	Phe416	π–π	62.14
8a	Phe384	π–π	12.29
8a	Tyr419	π–π	3.40
8a	Tyr211	π–π	1.80
8a	His252	π–π	0.50
8a	His212	π–π	0.10
8a	Lys359	π–cation	3.70
8a	Tyr419	π–cation	2.10
8a	His252	π–cation	0.90
8a	Leu401	Hydrophobic	19.48
8a	Leu420	Hydrophobic	13.39
8a	Val380	Hydrophobic	12.19
8a	Ile323	Hydrophobic	11.29
8a	Phe416	Hydrophobic	8.79
8a	Tyr419	Hydrophobic	7.19
8a	Pro400	Hydrophobic	3.60
8a	Ile412	Hydrophobic	3.40
8a	Phe384	Hydrophobic	2.90
8a	Pro366	Hydrophobic	0.40
8a	Ile363	Hydrophobic	0.20
TC8	Asn260	Hydrogen bond	28.67
TC8	His256	Hydrogen bond	14.39
TC8	Cys402	Hydrogen bond	0.20
TC8	Phe384	π–π	68.03
TC8	His212	π–π	20.18
TC8	His256	π–π	0.80
TC8	Phe416	π–π	0.20
TC8	Tyr211	π–π	0.10
TC8	Lys359	π–cation	1.80

Complex	Residue	Interaction type	Occupancy (%)
TC8	Phe384	Hydrophobic	21.08
TC8	Ile323	Hydrophobic	17.98
TC8	Leu420	Hydrophobic	17.18
TC8	Leu401	Hydrophobic	8.89
TC8	Val380	Hydrophobic	5.69
TC8	Ile363	Hydrophobic	4.60
TC8	Phe416	Hydrophobic	3.90
TC8	Tyr211	Hydrophobic	2.30
TC8	Pro262	Hydrophobic	2.20
TC8	Leu281	Hydrophobic	0.30
TC8	Tyr419	Hydrophobic	0.30
TC8	Pro400	Water bridge	39.16
TC8	Gln261	Water bridge	17.88
TC8	Ser399	Water bridge	13.29
TC8	His212	Water bridge	4.00
TC8	Val259	Water bridge	3.30
TC8	Asn260	Water bridge	3.10
TC8	His256	Water bridge	1.10
TC8	Gln387	Water bridge	0.10
TC8	Tyr211	Water bridge	0.10
TC8	Asn260	Metal-associated contact	0.10

Note. Occupancy was calculated as the percentage of the 1,001 analyzed trajectory frames containing at least one interaction of the specified subtype between the ligand and the indicated PDE7A residue. Different interaction subtypes involving the same residue are listed separately. The table includes all residue-specific interactions available in the exported Desmond contact files, including hydrogen bonds, hydrophobic contacts, π–π interactions, π–cation interactions, and, where available, water-bridge and metal-associated contacts. The corresponding Schrödinger interaction-fraction histograms and contact timelines in Supplementary Figures S2–S6 display the broader interaction categories detected by the Simulation Interactions Diagram. Interaction categories for which residue-level numerical export files were unavailable were not assigned quantitative occupancies in this table. Contacts below 10% are included for completeness but were considered transient.
